# Isotope Effect of Host Material on Device Stability of Thermally Activated Delayed Fluorescence Organic Light‐Emitting Diodes

**DOI:** 10.1002/smsc.202000057

**Published:** 2021-02-07

**Authors:** Xuelong Liu, Chin-Yiu Chan, Fabrice Mathevet, Masashi Mamada, Youichi Tsuchiya, Yi-Ting Lee, Hajime Nakanotani, Shinichiro Kobayashi, Masayuki Shiochi, Chihaya Adachi

**Affiliations:** ^1^ Center for Organic Photonics and Electronics Research (OPERA) Kyushu University 744 Motooka, Nishi Fukuoka 819-0395 Japan; ^2^ Institut Parisien de Chimie Moléculaire CNRS UMR 8232 Chimie des Polymères Sorbonne Université 4 place Jussieu 75005 Paris France; ^3^ International Institute for Carbon Neutral Energy Research (WPI-I2CNER) Kyushu University 744 Motooka, Nishi Fukuoka 819-0395 Japan; ^4^ Fukuoka i3-Center for Organic Photonics and Electronics Research (i3-opera) 5-14 Kyudai-shinmachi, Nishi-ku Fukuoka 819-0388 Japan

**Keywords:** deuteration of organic functional materials, isotope effects, operational stability, organic light-emitting diodes, thermally activated delayed fluorescence

## Abstract

Organic light‐emitting diodes (OLEDs) exhibiting thermally activated delayed fluorescence (TADF) have attracted great interest because of their excellent exciton harvesting ability in electroluminescence (EL). While TADF‐OLEDs show a high EL efficiency, the device operational stability is not necessarily satisfactory for commercial applications. Herein, the isotope effect of the host material on the device operational stability in TADF‐OLEDs is investigated. It is unveiled that the deuterated host, i.e., PYD2Cz‐***d***_***16***_, forms a denser film than that of the nondeuterated host, PYD2Cz, demonstrating enhanced stable amorphous nature and balanced carrier transport properties. In green TADF‐OLEDs, the PYD2Cz‐***d***_***16***_‐based OLED considerably lengthens the device operational stability of LT_95_ (95% of the initial luminance) to ≈140 h at an initial luminance of 1000 cd m^−2^, which is 1.7 times longer than that of PYD2Cz. Device stabilities of blue TADF‐OLEDs with PYD2Cz‐***d***_***16***_ as a host are also demonstrated with an enhancement of 2 times in LT_50_ at an initial luminance of 1000 cd m^−2^. It is suggested that the deuterated materials have a positive effect on the device stability in not only TADF‐OLEDs, but also all other OLEDs having fluorescence and phosphorescence emitters.

## Graphical abstract

A deuterated host (**PYD2Cz‐*d_16_***) is introduced in thermally activated delayed fluorescence organic light‐emitting diodes (TADF–OLEDs). Using deuterated host, the lifetime of TADF OLEDs is extended. Such improvements could be attributed to the noncrystalline and densely packed behaviors of film state in deuterated host, resulting in much more balanced carrier‐transporting properties in devices.

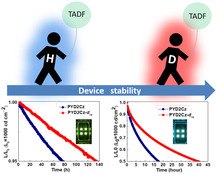

## 1 Introduction

The triplet exciton harvesting as delayed fluorescence by the aid of thermal energy, namely thermally activated delayed fluorescence (TADF), has attracted great interest in recent years because TADF can provide an ultimate electroluminescence (EL) quantum efficiency in organic light‐emitting diodes (OLEDs). When a small energy gap between lowest singlet excited state (*S*_1_) and triplet excited state (*T*_1_) is present in an organic molecule, the triplet excitons can be upconverted into singlet excitons through the reverse intersystem crossing (RISC). Using the molecule exhibiting TADF as the emitter in OLEDs, the internal EL quantum efficiency (IQE) of 100% can be achieved without the utilization of noble metals.^[^ ^1^, ^2^, ^3^, ^4^ ^]^ As the efficient TADF‐OLEDs were reported in 2012,^[^ ^2^ ^]^ numerous efforts have been put to improve the EL efficiency and color purity of EL; however, the device operational stabilities of TADF‐OLEDs remain as the main obstacle for commercialization of TADF‐OLEDs.^[^ ^5^, ^6^, ^7^, ^8^, ^9^, ^10^ ^]^

As the high density formation of triplets in an emitting layer (EML) during OLED operation would cause device degradation, many researchers have tried to extend the device operational lifetime using TADF emitters with a fast RISC rate constant (*k*_RISC_).^[^ ^7^, ^8^ ^]^ Furthermore, the fluorescent radiative decay rate constant (*k*_r_) of TADF emitters is also an important factor for the device stability as well.^[^ ^11^, ^12^ ^]^ In addition to the rational molecular design of TADF emitters for having a longer device operational lifetime, the choice of host materials should have a pronounced effect on device stability. In fact, it has been reported that the device operational stability of TADF or phosphorescent (Ph) OLEDs are significantly increasing when chemically stable materials are utilized as the host.^[^ ^9^, ^13^, ^14^, ^15^ ^]^ Numerous reports of rational molecular design of the host materials have pointed out the importance of balanced carrier transporting properties of host materials to expand a recombination zone and extend their device stability.^[^ ^16^, ^17^ ^]^

In fact, the device operational lifetime of the reported Ph‐OLED using deuterated host materials, in which hydrogen atoms are replaced by deuterium atoms, was improved; LT_50_ from 0.5 to 2 h.^[^ ^18^ ^]^ It is believed that the reason for extended device lifetime is originated from the heavier deuterium atom that may lead slower kinetic rate for unwarranted chemical reactions.^[^ ^18^, ^19^, ^20^, ^21^ ^]^ However, the mechanism of improved device stability of deuterated hosts in OLEDs is still unclear, and there is no report of using deuterated hosts in TADF‐OLEDs.

**Figure 1:**
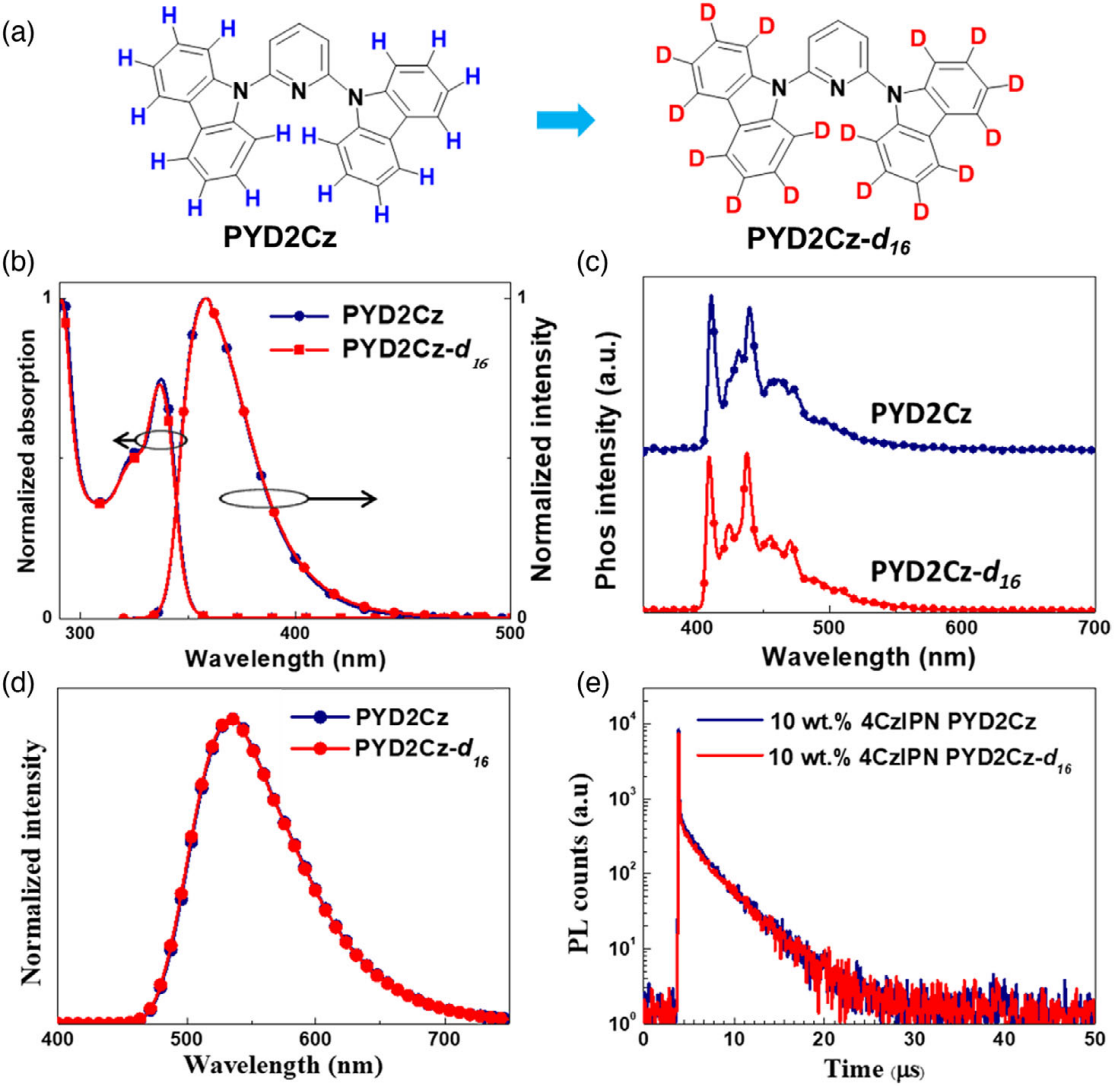
a) The chemical structures of **PYD2Cz** and **PYD2Cz‐** ***d***_***16***_. b) Absorption and PL spectra of **PYD2Cz** and **PYD2Cz‐** ***d***_***16***_ in toluene in 298 K (10^−5^ m). c) Phosphorescent spectra of **PYD2Cz** and **PYD2Cz‐** ***d***_***16***_ in toluene solution at 77 K. d) Emission spectra and e) decay curves of vacuum‐evaporated doped films with doping concentrations of 10 wt% of 4CzIPN in **PYD2Cz** and **PYD2Cz‐** ***d***_***16***_ hosts at excitation wavelength of 300 nm.

In this study, we introduced a deuterated host, namely2,6‐di(9H‐carbazol‐9‐yl‐)pyridine‐***d***_***16***_ (**PYD2Cz‐** ***d***_***16***_ **)**, in blue and green TADF‐OLEDs to investigate the isotope effect on the OLED performances, especially, the device operational stability (**Figure** **1**a). The photophysical properties of **PYD2Cz‐** ***d***_***16***_ are similar to that of nondeuterated **PYD2Cz**; however, the physical properties of their thin films are quite different. Importantly, **PYD2Cz‐** ***d***_***16***_ forms a denser film than that of the **PYD2Cz**. The operational stability (LT_95_) of the **PYD2Cz‐** ***d***_***16***_ **‐**based TADF‐OLED (green device) is 1.7‐times longer than that of the **PYD2Cz**‐based TADF‐OLED. Hole‐only and electron‐only devices (HODs/EODs) demonstrated that **PYD2Cz‐** ***d***_***16***_ possesses comparable hole transport and electron transport properties. The well‐balanced carrier transport properties of **PYD2Cz‐** ***d***_***16***_ can provide a broad recombination zone, hence increasing the LT_95_ in the OLEDs.

## 2 Results and Discussion

**PYD2Cz** and **PYD2Cz‐** ***d***_***16***_ were synthesized by nucleophilic substitution reaction in *N,N*‐dimethylformamide at 150 °C (Figure S1–S3, Supporting Information). To confirm the deuteration of the materials, infrared (IR) spectra of **PYD2Cz** and **PYD2Cz‐** ***d***_***16***_ were measured, as shown in Figure S4, Supporting Information); the peaks of aromatic C—H and C—D stretching can be found in 3042 and 2282 cm^−1^, respectively. To further confirm the structural packing of the **PYD2Cz** and **PYD2Cz‐** ***d***_***16***_, the single‐crystal X‐ray diffraction analyses were carried out (Table S1, S2, and Figure S5, Supporting Information). Fortunately, the crystal quality was high for both compounds (*R* = 3.65% for **PYD2Cz** and 3.63% for **PYD2Cz‐** ***d***_***16***_), making the comparison of parameters possible. Thermal properties of **PYD2Cz** and **PYD2Cz‐** ***d***_***16***_ were examined by differential calorimetry (DSC) under a nitrogen atmosphere (Figure S6, Supporting Information). On second heating, the thermograms of **PYD2Cz** and **PYD2Cz‐** ***d***_***16***_ both show a glass transition (*T*_g_) at 74.3 and 72.6 °C, respectively. On further heating, **PYD2Cz** presents a cold crystallization after the *T*_g_ and the compound does finally melt around 221 °C. Interestingly, **PYD2Cz‐** ***d***_***16***_ did not display cold crystallization and any melting processes in the temperature range of 0–240 °C. This absence of cold crystallization and melting processes demonstrates the strong amorphous character of **PYD2Cz‐** ***d***_***16***_ in bulk, and may be beneficial to the stable device operation even under high‐temperature when comparing with **PYD2Cz**.

The ultraviolet–visible (UV–vis) absorption and photoluminescence (PL) spectra of **PYD2Cz** and **PYD2Cz‐** ***d***_***16***_ in toluene (10^−5^ m) are shown in Figure 1b. The absorption and emission spectra of **PYD2Cz** and **PYD2Cz‐** ***d***_***16***_ were almost identical. The *T*_1_ energies of **PYD2Cz** and **PYD2Cz‐** ***d***_***16***_ were found to be 3.01 and 3.03 eV, respectively, which were determined from the highest energy vibronic peak of their phosphorescence spectra in toluene at 77 K (Figure 1c). The UV–vis absorption spectrum of **PYD2Cz‐** ***d***_***16***_ in the neat film is the same as that of **PYD2Cz**, but the PL emission peak of **PYD2Cz‐** ***d***_***16***_ in the neat film is slightly red shifted by 15 nm (Figure S7, Supporting Information), which indicated that the *π*‐interaction in **PYD2Cz‐** ***d***_***16***_ in the neat film would be stronger than that of the **PYD2Cz** film. In fact, the crystal structure analysis revealed a small difference of dihedral angles between pyridine and carbazole rings, and intermolecular distances (Figure S5, Supporting Information).

The highest occupied molecular orbital (HOMO) energy levels of **PYD2Cz** and **PYD2Cz‐** ***d***_***16***_ were estimated to be –6.05 and –6.07 eV, respectively (Figure S8, Supporting Information). The optical energy gaps were the same for both hosts (3.36 eV), and the lowest unoccupied molecular orbital (LUMO) energy levels of **PYD2Cz** and **PYD2Cz‐** ***d***_***16***_ were calculated to be –2.69 and –2.71 eV, respectively. The *S*_1_ and *T*_1_ of both hosts were high enough to confine the singlet and triplet excitons of green TADF emitter such as (4s,6s)‐2,4,5,6‐tetra(9H‐carbazol‐9‐yl)isophthalonitrile (4CzIPN) (*T*_1_ **= **2.67 eV, *S*_1_ = 2.71 eV).

We then investigated the photophysical properties of vacuum‐evaporated thin films of 4CzIPN doped in **PYD2Cz** and **PYD2Cz‐** ***d***_***16***_. With a doping concentration of 10 wt% of 4CzIPN, both doped films showed identical PL peaks at 534 nm (Figure 1d). The transient PL decay curves of both doped films are shown in Figure 1e, in which the prompt and delayed components were clearly observed without any noticeable difference in these films, indicating almost no isotope effect on the PL properties in the co‐deposited films. The detailed photophysical properties of both materials in solutions and in doped films are shown in **Table** **1** and **2**. Note that 4CzIPN doped in a **PYD2Cz‐** ***d***_***16***_ host showed slightly higher PL quantum efficiency than that of a **PYD2Cz** host, probably due to the lower vibration energy of C—D bond.

**Table 1:** Physical properties of **PYD2Cz** and **PYD2Cz‐** ***d***_***16***_

	Fluoa) *λ*_max_ [nm]	Fluob) *λ*_max_ [nm]	Phosc) *λ*_max_ [nm]	*T*_1_ d) [eV]	HOMOe) [eV]	LUMOf) [eV]
**PYD2Cz**	359	375	411	3.01	−6.05	−2.69
**PYD2Cz‐** ***d***_***16***_	358	390	409	3.03	−6.07	−2.71

a) Emission peak measured in 10^−5^ m toluene solutions at room temperature;

b) Measured on neat film samples at room temperature;

c) Measured in 10^−5^ m toluene solutions at 77 K;

d) Measured in solution at 77 K;

e) Measured on film samples using Riken‐Keiki AC‐3;

f) Estimated from the HOMO values and optical bandgap.

**Table 2:** Physical properties of **PYD2Cz** or **PYD2Cz‐** ***d***_***16***_ doped films (PL_max_: PL peak; Φ_PL_: PL quantum yield; *τ*_p_: prompt emission decay lifetime; *τ*_d_: delayed emission decay lifetime; Φ_P_: efficiency of prompt emission; Φ_d_: efficiency of delayed emission; *k*_ISC_: intersystem crossing rate; *k*_RISC_: reversed intersystem crossing rate)

	PL_max_ [nm]	Φ_PL_ [%]	*τ*_p_ [ns]	*τ*_d_ [μs]	Φ_p_ [%]	Φ_d_ [%]	*k*_ISC_ [10^7^ s^−1^]	*k*_RISC_ [10^6^ s^−1^]
10 wt% 4CzIPN **PYD‐2Cz**	534	94	27.4	2.7	20	74	2.87	1.74
10 wt% 4CzIPN **PYD2Cz‐** ***d***_***16***_	534	98	27.0	2.8	21	77	2.87	1.69

**Figure 2:**
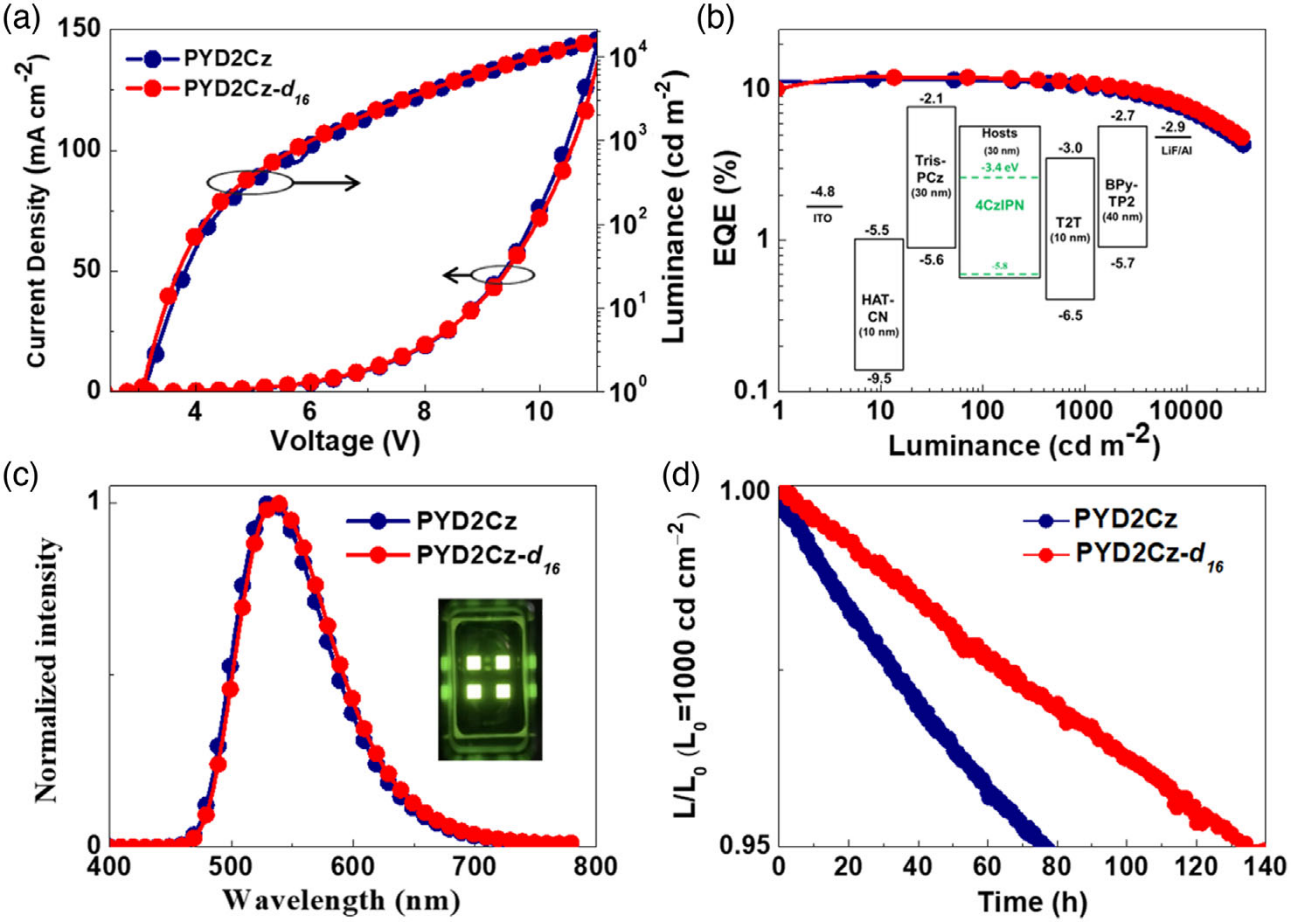
Device performance of **PYD2Cz** and deuterated **PYD2Cz‐** ***d***_***16***_‐based green TADF‐OLED devices. a) Current density–voltage–luminance (*J*–*V*–*L*) curves. b) External quantum efficiency versus luminance. Inset: Energy‐level diagram of the fabricated devices. **c**) EL spectra of devices. d) Normalized luminance versus time (at an initial luminance of 1000 cd m^−2^).

To evaluate the isotope effect of host material on the device operational stability, green TADF‐OLED devices were fabricated with a configuration as follows: ITO/HAT‐CN (10 nm)/Tris‐PCz (30 nm)/10 wt%‐4CzIPN: **PYD2Cz** or **PYD2Cz‐** ***d***_***16***_ (30 nm)/T2T (10 nm)/BPy‐TP2 (40 nm)/LiF (0.8 nm)/Al (70 nm) and details are shown in **Figure** **2**. In the device, HAT‐CN, Tris‐PCz, T2T, BPy‐TP2, and LiF were used as a hole‐injection layer (HIL), a hole transport layer (HTL), a hole blocking layer (HBL), an electron‐transporting layer (ETL), and an electron‐injection layer (EIL), respectively (Figure 2b, inset). The EL spectrum in both devices, i.e., Commission Internationale de l'Éclairage (CIE) *x*, *y* coordinates of (0.35,0.59), and (0.36,0.58), were superimposable with their PL spectra, indicating the well‐confinement of 4CzIPN excitons in the EMLs. Although both **PYD2Cz** and **PYD2Cz‐** ***d***_***16***_ devices exhibited similar maximum external EL quantum efficiencies (EQEs) of 11.7% and 12.4%, respectively, their device stabilities were totally different. The LT_95_ of the device based on **PYD2Cz‐** ***d***_***16***_ showed enhanced device stability of 134 h, as shown in Figure 2d, which is 1.7‐times longer than that of the **PYD2Cz**‐based OLED (LT_95_ = 77 h) at an initial luminance of 1000 cd m^−2^. Green devices were evaluated at a constant current density of 1.4 mA cm^−2^, in which the enhanced device stability was still displayed when **PYD2Cz‐** ***d***_***16***_ was used as the host material (Figure S9, Supporting Information).

**Figure 3:**
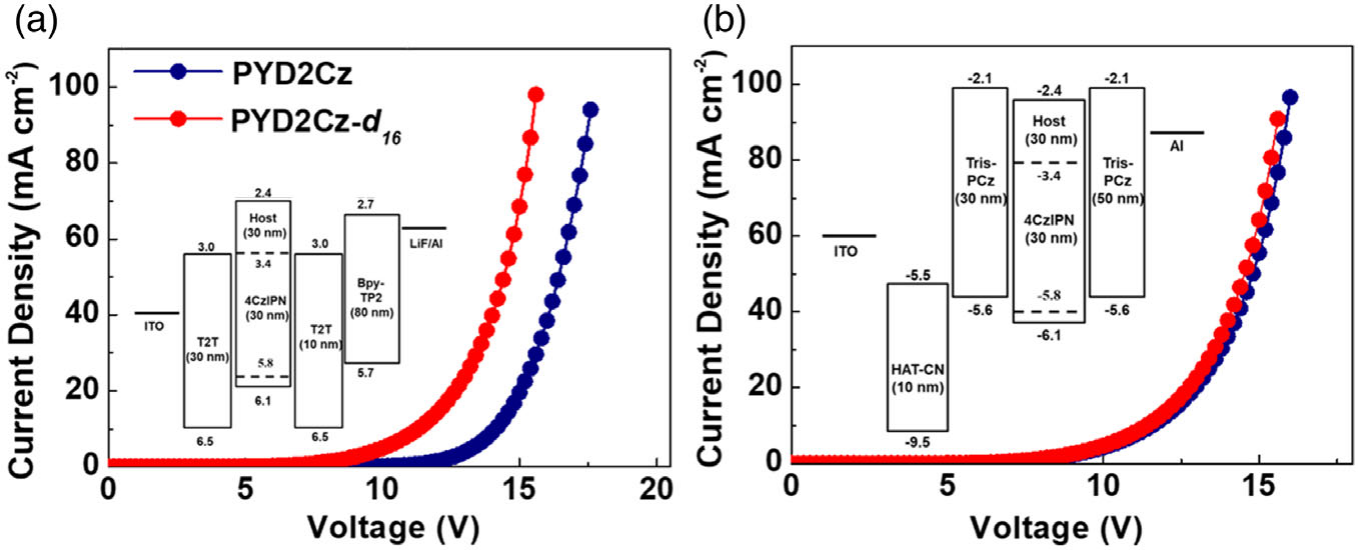
Current density–voltage (*J*–*V*) characteristics of a) EODs and b) HODs for **PYD2Cz** and **PYD2Cz‐** ***d***_***16***_ doped with 10 wt% 4CzIPN‐based. Inset: energy diagram of the a) EODs and b) HODs.

Several factors should be considered to explain the superior device lifetime of **PYD2Cz‐** ***d***_***16***_ based OLEDs. For example, it would be probable that **PYD2Cz‐** ***d***_***16***_ does not seriously aggregate during continuous driving of the OLEDs, even though Joule heating during operation, due to its noncrystallized state at a temperature above the glass transition temperature, which was confirmed both in DSC measurement and atomic force microscopy images (Figure S6 and S10, Supporting Information). The **PYD2Cz** film showed an amorphous film before heating (Figure S10a, Supporting Information), but the aggregates were found after heating at 130 °C (Figure S10b, Supporting Information); however, **PYD2Cz‐** ***d***_***16***_ was found to be a noncrystalline film before (Figure S10c, Supporting Information) and after heating at 130 °C (Figure S10d, Supporting Information). In addition, the more kinetically stable C—D bond in **PYD2Cz‐** ***d***_***16***_ than the C—H bond in **PYD2Cz** would be considered as a reason for the enhanced device lifetime. Here, we point out the effect of the isotope substitution in the **PYD2Cz** on charge transport properties. The HODs and EODs were fabricated using two hosts with the device configuration shown in **Figure** **3**. It was found that the **PYD2Cz‐** ***d***_***16***_‐based HODs/EODs displayed well‐balanced charge transport properties than that of the **PYD2Cz**‐based device. At 12 V, in the **PYD2Cz‐** ***d***_***16***_, the ratio of current densities of the EODs to the HODs was close to 1, whereas it was only 0.12 in the **PYD2Cz**‐based HODs/EODs. To explain this behavior, the thin‐film densities (*ρ*) of **PYD2Cz** and **PYD2Cz‐** ***d***_***16***_ were determined, and it is found that the **PYD2Cz‐** ***d***_***16***_ film (*ρ* = 1.189 ± 0.006 g cm^−3^) was denser than the **PYD2Cz** film (*ρ* = 1.146 ± 0.014 g cm^−3^) (Figure S11 and see Supporting Information for the detailed experimental method). Note that the crystal structure data also support a higher density for **PYD2Cz‐** ***d***_***16***_ due to smaller volume (Table S1 and S2, Supporting Information). The difference of the density is not so large, but it has been reported that the small difference critically affects the carrier transport properties in organic thin films.^[^ ^22^ ^]^ The denser thin film of **PYD2Cz‐** ***d***_***16***_ thus could explain the more balanced carrier transporting properties of **PYD2Cz‐** ***d***_***16***_, which was also consistent with the more red shifted PL emission (Figure S7, Supporting Information). The crystal structures indicated a stronger interaction between pyridine rings for **PYD2Cz‐** ***d***_***16***_ than **PYD2Cz**, which is considered to be a path for the electron transport (Figure S5, Supporting Information). We suppose that the balanced carrier transporting properties in **PYD2Cz‐** ***d***_***16***_ resulted in a broader recombination zone, hence a better device operational stability.^[^ ^5^ ^]^

**Figure 4:**
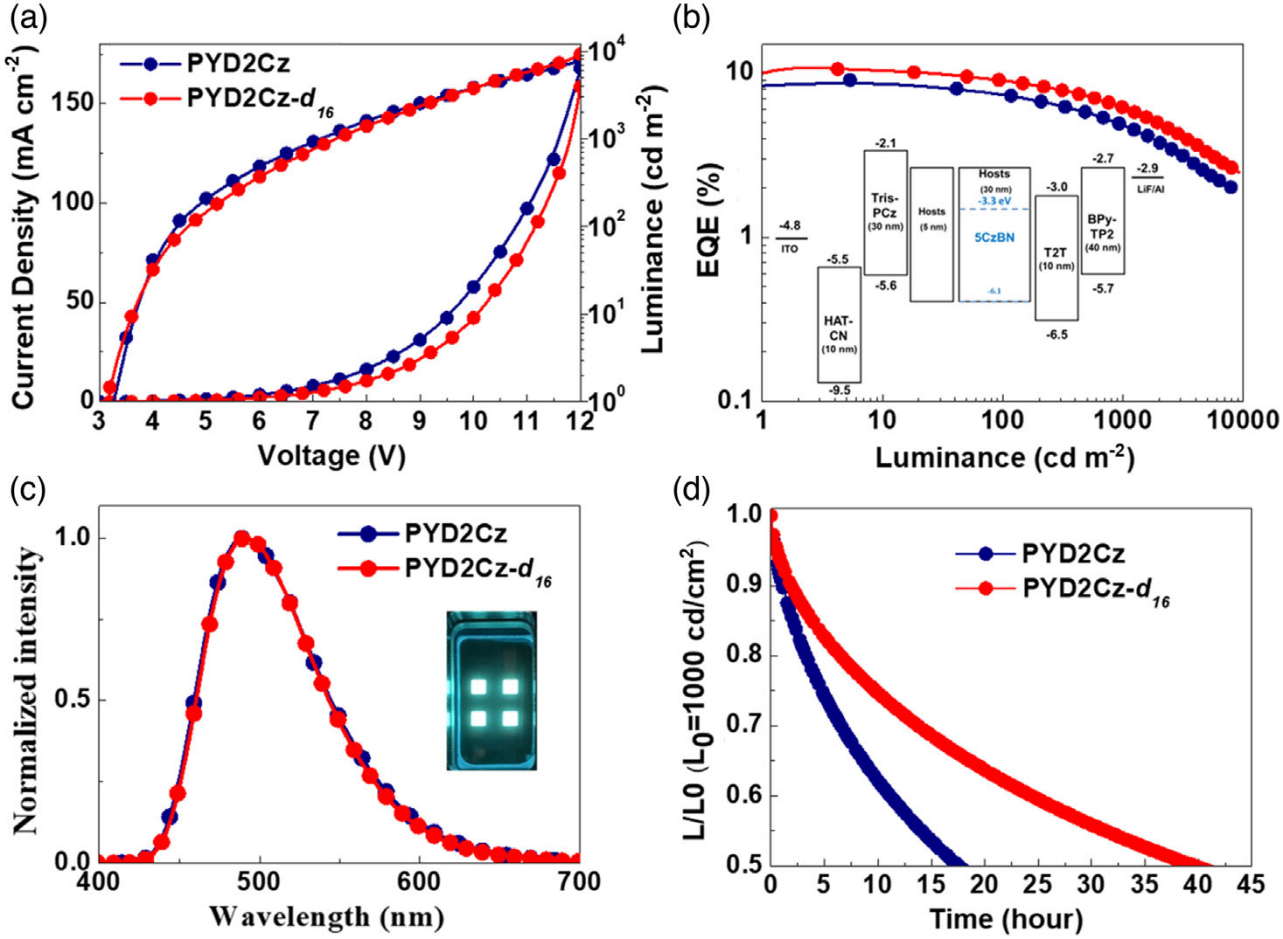
Device performance of **PYD2Cz** and deuterated **PYD2Cz‐** ***d***_***16***_‐based blue TADF‐OLED devices. a) *J*–*V*–*L* curves. b) External quantum efficiency versus luminance. Inset: Energy‐level diagram of the fabricated devices. **c)** EL spectra of devices. d) Normalized luminance versus time (at an initial luminance of 1000 cd m^−2^).

In addition to the green TADF‐OLEDs based on 4CzIPN, we further fabricated blue TADF‐OLEDs containing an EML of 10 wt%‐penta(9H‐carbazol‐9‐yl)benzonitrile (5CzBN) emitter in a **PYD2Cz** or **PYD2Cz‐** ***d***_***16***_ host. The device configuration was shown in the inset of **Figure** **4**b. The device performance is shown in Figure 4. Similar to the green TADF‐OLEDs, both **PYD2Cz** and **PYD2Cz‐** ***d***_***16***_ **‐**based OLEDs showed identical emission peak maxima of 491 nm and similar maximum EQEs of 9.1% and 11.2%, respectively. Nonetheless, a twofold‐enhanced device stability (LT_50_ at an initial luminance of 1000 cd m^−2^) was found (improved from 17 to 40 h) when **PYD2Cz‐** ***d***_***16***_ was used as the host material. Meanwhile, it was noted that the current densities for two devices are different at 1000 cd m^−2^. Therefore, device stabilities of both blue devices were evaluated at a constant current density of 5.4 mA cm^−2^, in which enhanced device stability was still displayed when **PYD2Cz‐** ***d***_***16***_ was used as the host material (Figure S12, Supporting Information). These proved that deuterated host **PYD2Cz‐** ***d***_***16***_, which has a high *T*_1_ level with well‐balanced charge carrier transport ability, is considered to be a potential candidate as a universal host for TADF‐OLEDs to enhance the device operational stability.

## 3 Conclusion

In conclusion, we revealed the isotopic effect of host materials, i.e., **PYD2Cz** and **PYD2Cz‐** ***d***_***16***,_ on the OLED performances in the TADF–OLEDs. Although the photophysical properties of both hosts are almost identical, it was found that the physical properties of hosts in the thin‐film states were appreciably different. Deuterated host (**PYD2Cz‐** ***d***_***16***_) showed noncrystalline and densely packed behaviors in a film state that was different from the nondeuterated host of **PYD2Cz**. Moreover, green TADF–OLEDs based on **PYD2Cz‐** ***d***_***16***_ displayed a more balanced carrier transporting properties than that of **PYD2Cz**, which resulted in better device stability with a 1.7 times longer in LT_95_ of 134 h at an initial luminance of 1000 cd m^−2^. The enhancement of the device operation lifetime by the deuterated host was also confirmed in the blue TADF–OLEDs. Our work could provide the explanations on why hydrogen/deuterium isotope effect improves the stability of OLEDs in the view of thermal and carrier transport properties. We, thus, believe that our study would give insight on achieving stable TADF/Ph/fluorescence‐OLEDs through the deuteration of organic functional materials.

## Conflict of Interest

The authors declare no conflict of interest.

## Supporting information

Supplementary Material
