# Supplementary material for: Isotope Effect of Host Material on Device Stability of Thermally Activated Delayed Fluorescence Organic Light‐Emitting Diodes
Source: Small Sci. 2021 Feb 7;1(4):2000057. doi: 10.1002/smsc.202000057 (PMC11936013; doi:10.1002/smsc.202000057)
Supplement: Supplementary file 1 — Supplementary Material [file SMSC-1-2000057-s001.docx]

Supporting Information

Isotope Effect of Host Material on Device Stability of Thermally Activated Delayed Fluorescence Organic Light-Emitting Diodes

Xuelong Liu, Chin-Yiu Chan*, Fabrice Mathevet, Masashi Mamada, Youichi Tsuchiya, Yi-Ting Lee, Hajime Nakanotani, Shinichiro Kobayashi, Masayuki Shiochi, and Chihaya Adachi*

**Methods**

**General**

**PYD2Cz** was purchased from Lumtec. Corp. All reagents were used as received from commercial sources and were used without further purification. Chromatographic separations were carried out using silica gel (200–300 nm). The two materials investigated in this paper were synthesized by following the procedures described below. **PYD2Cz** and **PYD2Cz-*d_16_*** were purified twice by temperature gradient vacuum sublimation. ^1^H nuclear magnetic resonance (NMR) spectra was obtained in CD_2_Cl_2_ with a Bruker Biospin Avance-III 500 NMR spectrometer at ambient temperature. Chemical shifts (δ) are given in parts per million (ppm) relative to tetramethylsilane (TMS; δ = 0) as the internal reference. Mass spectra were measured in positive-ion atmospheric-pressure chemical ionization (APCI) mode on a Waters 3100 mass detector. The purity of **PYD2Cz-*d_16_*** was confirmed by the high performance liquid chromatography (HPLC) method. The experimental condition of HPLC is as follows; Column: InertSustain C18 (2.1 mm × 150 mm), Carrier: Acetonitrile: water = 83: 17, Rate: 0.2 ml/min, Temperature: 40 °C, Det.: 254 nm. AFM images of **PYD2Cz** and **PYd2Cz-*d_16_*** neat film were investigated by tapping mode AFM (nGauge AFM).

**Photophysical measurements**

Toluene solutions containing two materials (10^–5^M) were prepared to investigate their absorption and photoluminescence characteristics in the solution state. Thin-film samples (10% wt- doped in mCBP) were deposited on quartz glass substrates by vacuum evaporation to study their exciton confinement properties in the film state. Ultraviolet–visible absorption (UV-vis) and photoluminescence (PL) spectra were recorded on a Perkin-Elmer Lambda 950 KPA spectrophotometer and a FP-6500 fluorescence spectrophotometer. Phosphorescent spectra were recorded on a JASCO FP-6500 fluorescence spectrophotometer at 77 K. The optical energy gaps (S_1_) determined from the absorption edges of thin films. A UV photoelectron emission spectrometer (Riken Keiki AC-3) was used determine the HOMO energy levels of the compounds in the neat films. Absolute PL quantum yields were measured on a Quantaurus-QY measurement system (C11347-11, Hamamatsu Photonics) under nitrogen flow and all samples were excited at 360 nm. The transient PL decay characteristics of solution and doped film samples at room temperature were recorded using a Quantaurus-Tau fluorescence lifetime measurement system (C11367-03, Hamamatsu Photonics). FT-IR spectra were measured using the KBr pellet method on a JASCO FT/IR-6000 infrared spectrophotometer.

**Thermal properties**

Thermal gravimetry-differential thermal analysis (TG-DTA) was performed by Bruker TG-DTA 2400SA with a heating rate of 10 ^o^C min^–1^ under nitrogen atmosphere. Differential scanning calorimetry (DSC) analysis was performed by Netzsch DSC204 Phoenix calorimeter at a scanning rate of 5 °C min^–1^ under N_2_ atmosphere.

**Device fabrication and measurements**

The OLEDs were fabricated by vacuum deposition process without exposure to ambient air. After fabrication, the devices were immediately encapsulated with glass lids using epoxy glue in a nitrogen-filled glove box (O_2_~0.1ppm, H_2_O~0.1ppm). The indium–tin oxide surface was cleaned ultrasonically and sequentially with acetone, isopropanol and deionized water, then dried in an oven, and finally exposed to ultraviolet light and ozone for about 10 min. Organic layers were deposited at a rate of 1 Å/s. Subsequently, Liq and Al were deposited at 0.3 and 1 Å/s, respectively. The device area is ~ 0.04 cm^2^. The EQE and *J-V-L* measurements were performed using a Keithley 2400 source meter and an absolute external quantum efficiency (EQE) measurement system (C9920-12, Hamamatsu Photonics, Japan). For the device lifetime tests, the luminance and EL spectra of the driving devices in the normal direction were measured using a luminance meter (SR-3AR, TOPCON, Japan) under constant current density driving conditions with an initial luminance of 1000 cd m^−2^.

**X-ray**

X-ray diffraction data for **PYD2Cz** and **PYD2Cz-*d_16_*** were collected on a Rigaku AFC HyPix-6000 diffractometer with Mo-Kα radiation (λ = 0.71075 Å) at 100 K. Single crystals suitable for X-ray analysis were grown by sublimation. The sublimation zone temperature was set at 200 °C and constant flow of pure nitrogen carrier gas with the pressure was kept at 0.1 bar. After 24 h plate-like crystals were formed in the crystallization zone. Data collection, cell refinement, and data reduction were carried out using the software CrysAlisPro. The structure was solved by direct methods using the program SHELXT and refined by full-matrix least squares methods on *F*^2^ using SHELXL-2014.^[1]^ All materials for publication were prepared using the software Olex2.^[2]^ All non-hydrogen atoms were refined anisotropically. The positions of all hydrogen atoms were calculated geometrically and refined as a riding model. Crystallographic data have been deposited with Cambridge Crystallographic Data Centre (CCDC): Deposition numbers CCDC 2036278-2036279. Copies of the data can be obtained free of charge via http://www.ccdc.cam.ac.uk/conts/retrieving.html.

**Table S1** Crystal data for **PYD2Cz**

| Identification code | PYD2Cz |
| --- | --- |
| Empirical formula | C_29_H_19_N_3_ |
| Formula weight | 409.47 |
| Temperature | 100 K |
| Wavelength | 0.71075 Å |
| Crystal system | Monoclinic, |
| Space group | *P*2_1_/c |
| Unit cell dimensions | *a* = 13.0824(4) *α* = 90 |
|  | *b* = 19.0866(6) *β* = 106.150(4) |
|  | *c* = 8.5518(3) *γ* = 90 |
| Volume | 2051.10(12) |
| *Z* | 4 |
| Density (calculated) | 1.326 g/cm^3^ |
| Absorption coefficient | 0.079 mm^−1^ |
| *F*(000) | 856 |
| Crystal size | 0.35 × 0.23 × 0.13 mm^3^ |
| Theta range for data collection | 2.700 to 27.483°. |
| Index ranges | −16<=h<=16, −23<=k<=24, −11<=l<=10 |
| Reflections collected | 23535 |
| Independent reflections | 4692 [R(int) = 0.0287] |
| Completeness to theta = 27.483° | 99.84 |
| Absorption correction | Gaussian |
| Max. and min. transmission | 1.000 and 0.556 |
| Refinement method | Full-matrix least-squares on *F*^2^ |
| Data / restraints / parameters | 4692 / 0 / 289 |
| Goodness-of-fit on *F*^2^ | 1.058 |
| Final R indices [*I*>2sigma(*I*)] | *R*_1_ = 0.0365, *wR*_2_ = 0.0933 |
| R indices (all data) | *R*_1_ = 0.0425, *wR*_2_ = 0.0969 |
| Largest diff. peak and hole | 0.275 and −0.256 e.Å^−3^ |

**Table S2** Crystal data for **PYD2Cz-*d_16_***

| Identification code | PYD2Cz-*d_16_* |
| --- | --- |
| Empirical formula | C_29_H_3_D_16_N_3_ |
| Formula weight | 425.57 |
| Temperature | 100 K |
| Wavelength | 0.71075 Å |
| Crystal system | Monoclinic, |
| Space group | *P*2_1_/c |
| Unit cell dimensions | *a* = 13.0807(4) *α* = 90 |
|  | *b* = 19.0973(5) *β* = 106.108(3) |
|  | *c* = 8.5270(3) *γ* = 90 |
| Volume | 2046.47(11) |
| *Z* | 4 |
| Density (calculated) | 1.381 g/cm^3^ |
| Absorption coefficient | 0.079 mm^−1^ |
| *F*(000) | 856 |
| Crystal size | 0.339 × 0.25 × 0.077 mm^3^ |
| Theta range for data collection | 2.133 to 27.481°. |
| Index ranges | −16<=h<=16, −22<=k<=24, −10<=l<=11 |
| Reflections collected | 22080 |
| Independent reflections | 4678 [R(int) = 0.0286] |
| Completeness to theta = 27.481° | 99.86 |
| Absorption correction | Gaussian |
| Max. and min. transmission | 1.000 and 0.669 |
| Refinement method | Full-matrix least-squares on *F*^2^ |
| Data / restraints / parameters | 4678 / 0 / 289 |
| Goodness-of-fit on *F*^2^ | 1.050 |
| Final R indices [*I*>2sigma(*I*)] | *R*_1_ = 0.0363, *wR*_2_ = 0.0911 |
| R indices (all data) | *R*_1_ = 0.0425, *wR*_2_ = 0.0949 |
| Largest diff. peak and hole | 0.260 and −0.261 e.Å^−3^ |

**Thin film density measurement**

The measure method for film density is based on previous report.^[3]^ Neat films of **PYD2Cz** and **PYD2Cz-*d_16_*** deposited on Si substrate (~500 nm), the thicknesses of the neat films were determined using Variable angle spectroscopic ellipsometry (VASE). After that, the neat films were dissolved in 5 ml toluene and absorption spectra were measured with a spectrometer (LAMBDA 950, PerkinElmer). Comparing the absorbance with the calibration curves in Fig. S9, the film densities were calculated by dividing the mass by the volume.

**Synthesis**

**PYD2Cz-*d_16_***

Under nitrogen atmosphere, 9*H*-carbazole-d_8_ (2 mmol) was dissolved in dry *N*,*N*-dimethylformamide (30 mL) in a two-neck round-bottom flask equipped with a condenser. The reaction mixture was cooled to 0 °C, then NaH (2 mmol) was added. The reaction mixture was slowly warmed to room temperature and stirred for half an hour. After that, 2,6-difluoropyridine (1 mmol) was added and the reaction was heated to 150 °C for 16 hours. The reaction was quenched with water and the precipitate was filtered off. The crude product was purified by column chromatography. Yield: 341 mg (80%). ^1^H NMR (500 MHz, CD_2_Cl_2_, 298 K, relative to Me_4_Si): δ = 8.24 (t, 1H, *J* = 8Hz), 7.72 (d, 2H, *J* = 8 Hz). MS (APCI) calcd. For C_29_H_3_D_16_N_3_: m/*z* = 425.8; found: 426.4 [M]^+^. Purity (HPLC): 99.74%.

**Figure S1.** Synthetic step of **PYD2Cz-*d_16_***.

**Figure S2.** ^1^H NMR of **PYD2Cz-*d_16_***.

**Figure S3.** Chromatogram of PYD2Cz-*d16* by HPLC for purity check.


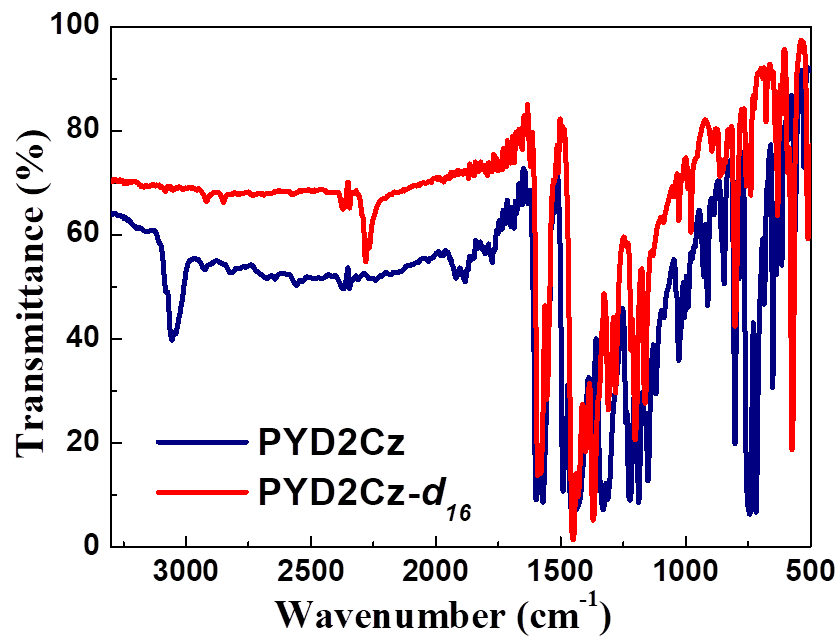


**Figure S4.** IR spectra of **PYD2Cz ­**and **PYD2Cz-*d_16_***.


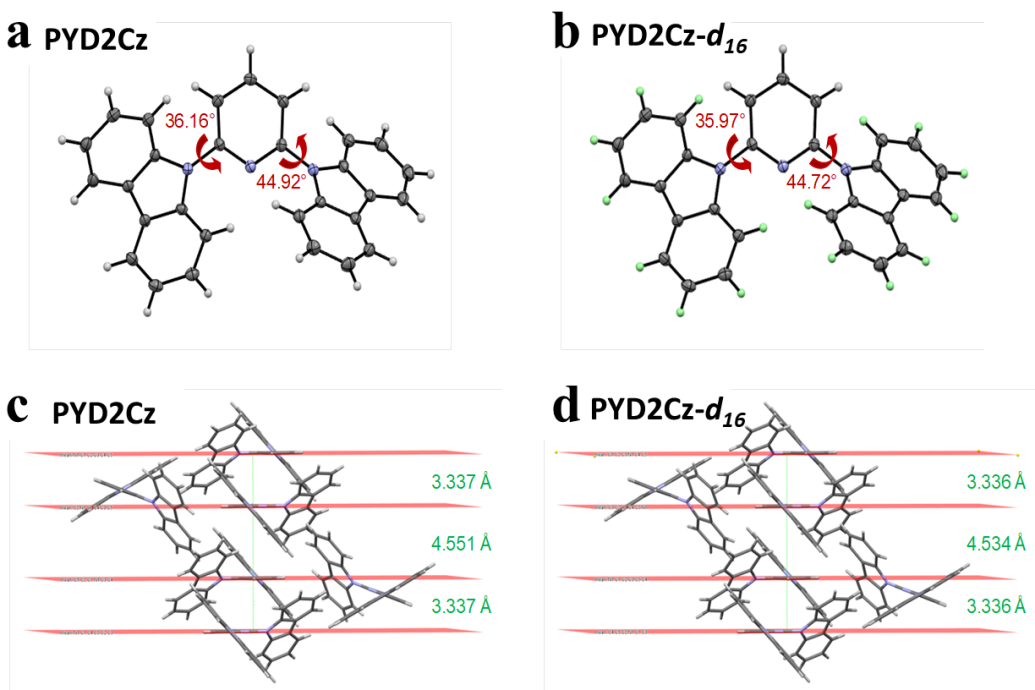


**Figure S5.** Single crystal structures of (a) **PYD2Cz ­**and (b) **PYD2Cz-*d_16_***. Packing structure and the distance between pyridine rings for (c) **PYD2Cz ­**and (d) **PYD2Cz-*d_16_***.


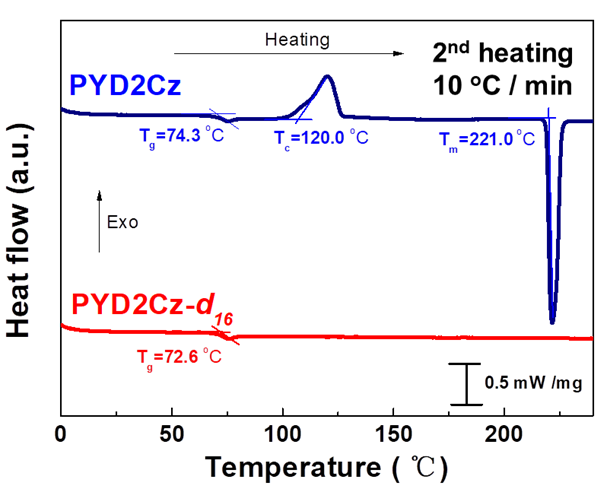


**Figure S6.** DSC curves of **PYD2Cz ­**and **PYD2Cz-*d_16_*** in 2^nd^ heating, in which *T*_g_, *T*_c_ and *T*_m_ are glass transition temperture, cold crystallization temperature and melting point, respectively.


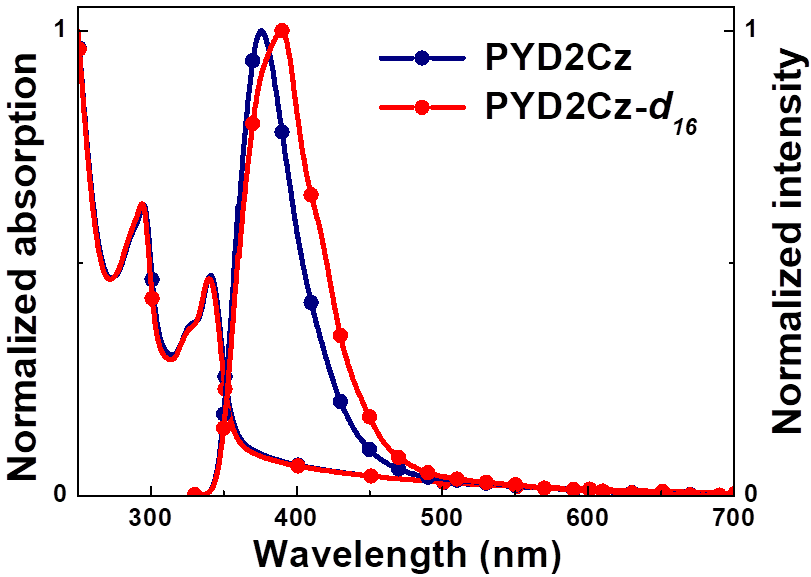


**Figure S7.** Absorption and photoluminescence spectra of **PYD2Cz ­**and **PYD2Cz-*d_16_*** in neat films.


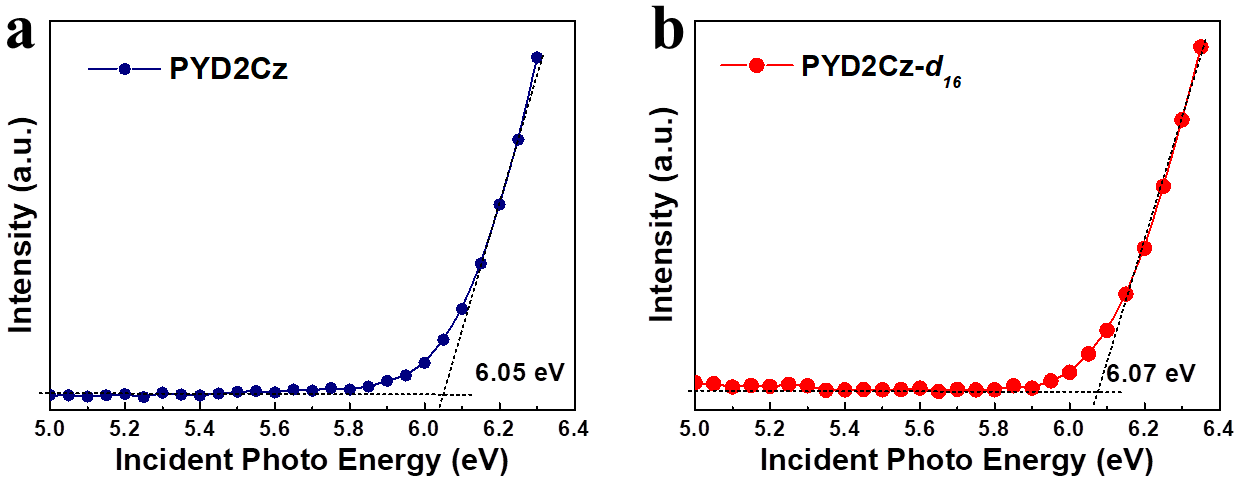


**Figure S8.** Photoelectron yield spectral measurements of (a) **PYD2Cz** and (b) **PYD2Cz-*d_16_*** neat film under nitrogen atmosphere.


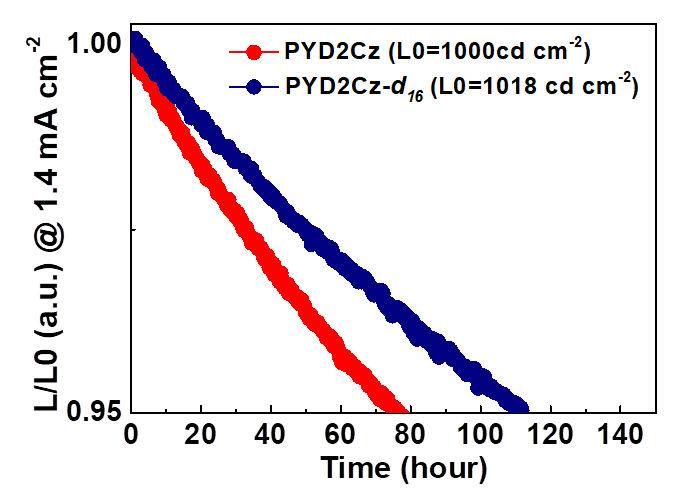


**Figure S9.** Normalized luminance versus time characteristics of **PYD2Cz ­**and **PYD2Cz-*d_16_*** based green TADF-OLED devices at a constant current density of 1.4 mA cm^-2^.


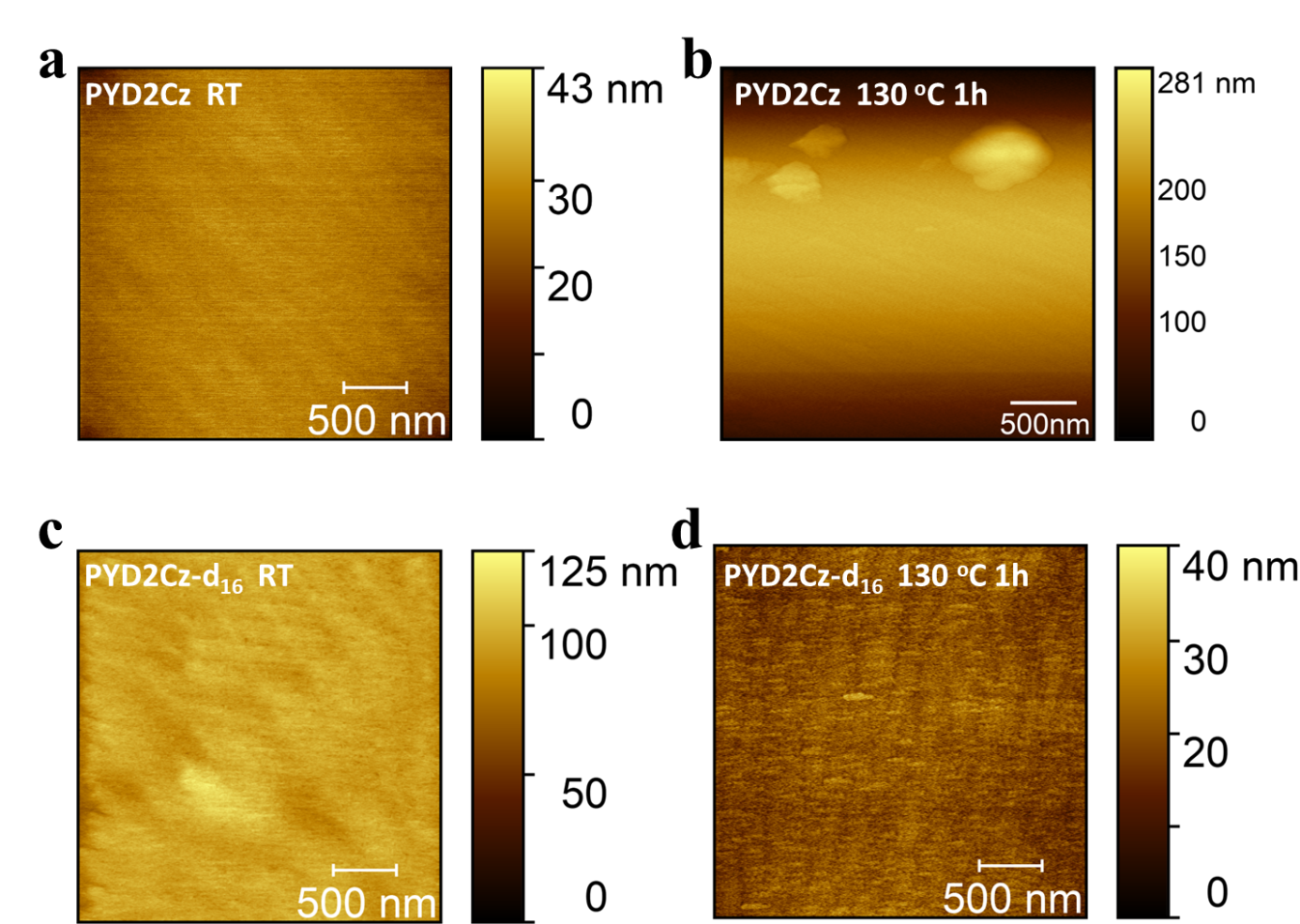


**Figure S10.** AFM images for **PYD2Cz** and **PYD2Cz-*d_16_***. Images were measured before annealing for **PYD2Cz** (a) and **PYD2Cz-*d_16_*** (c) neat films and after heating of 1h under 130 ^o^C for **PYD2Cz** (b) and **PYD2Cz-*d_16_*** (d) neat films.


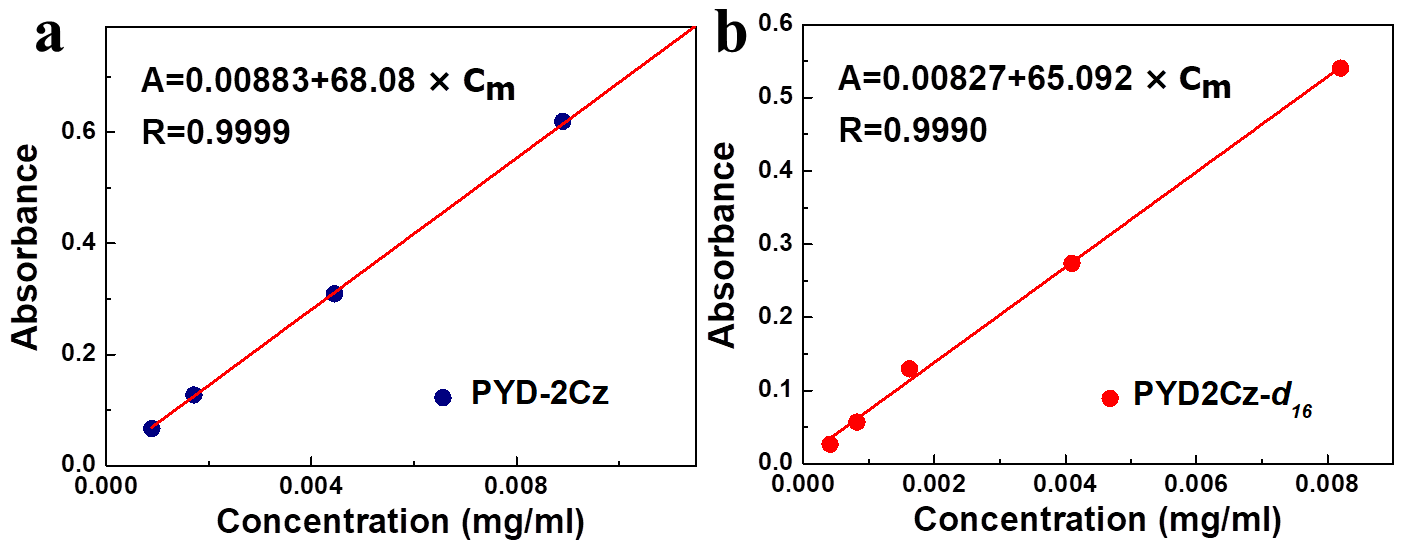


**Figure S11.** Calibration curves of absorption coefficient against concentration of (a) **PYD2Cz ­**and (b) **PYD2Cz-*d_16_*** in toluene.


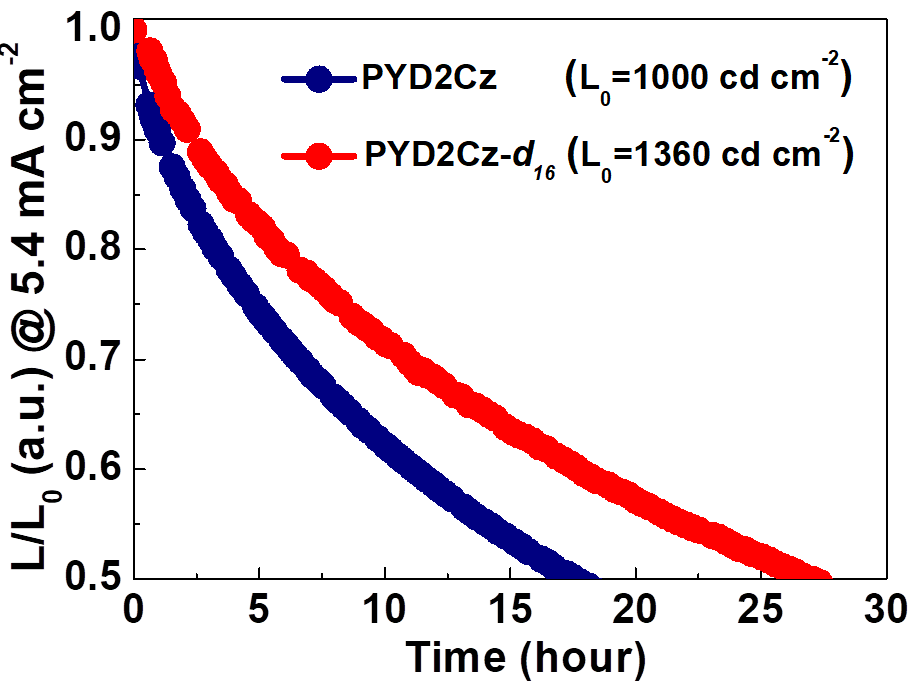


**Figure S12.** Normalized luminance versus time characteristics of **PYD2Cz ­**and **PYD2Cz-*d_16_*** based blue TADF-OLED devices at a constant current density of 5.4 mA cm^-2^.

**References**

[1] G. M. Sheldrick, *Acta Cryst.* **2015**, A71, 3.

[2] O. V. Dolomanov, L. J. Bourhis, R. J. Gildea, J. A. K. Howard, H. Puschmann, *J. Appl. Cryst*. **2009**, 42, 339.

[3] H. F. Xiang, Z. X. Xu, V.A.L. Roy, C. M. Che, P. T. Lai, *Rev. Sci. Instrum*. **2007**,

78, 034104.
